# A novel, simple near-infrared thoracoscopic technique by a particular route for locating lung nodules

**DOI:** 10.3389/fonc.2023.1278563

**Published:** 2023-10-10

**Authors:** Zhengjun Li, Mozhu Xia, Chang Liu, Liwei Xie, Tao Wang, Yi Ren

**Affiliations:** ^1^ Department of Thoracic Surgery, Shenyang Chest Hospital, Shenyang, China; ^2^ Department of Operation Room, First Affiliated Hospital, China Medical University, Shenyang, China; ^3^ Department of Radiology, Shenyang Chest Hospital, Shenyang, China; ^4^ Department of Anesthesia, Shenyang Chest Hospital, Shenyang, China

**Keywords:** fluorescence, near-infrared, lung cancer, image-guided surgery, indocyanine green

## Abstract

**Background:**

The localization of pulmonary nodules prior to thoracoscopic surgery remains challenging for thoracic surgeons, especially for those nodules that are not visible or palpable on the lung surface. Our study is a simple and effective minimally invasive method using indocyanine green through a special pathway to locate pulmonary nodules and fluorescence thoracoscopic surgery.

**Methods:**

Thoracoscopic surgery was performed for 18 undiagnosed peripheral non-solid nodules no larger than 2 cm after location. After 0.3 mg/kg indocyanine green was injected through the peripheral vein, the puncture needle was pulled out after it reached approximately 1 cm of the pulmonary parenchyma near the nodules. This was followed by transfer to the operating room. The nodule was initially localized by using a near-infrared thoracoscope to visualize indocyanine green fluorescence. Then, thoracoscopic resection was performed.

**Results:**

Eighteen patients received this special and simple localization method, and underwent near-infrared, image-guided, video-assisted thoracoscopic surgery resection. Median computed tomography (CT) tumor size was 1.2 cm. Median depth from the pleural surface is 1.6 cm (range, 0.1–4.6 cm). The median time of CT-guided intervention was 12 min. The duration of thoracoscopic surgery was 67 min. Indocyanine green fluorescence was clearly identified in 17 of 18 patients (94.4%). The surgical margins were all negative on final pathology. The final diagnoses included 17 primary lung cancers, and 1 benign lung tumor.

**Conclusions:**

CT-guided single puncture of indocyanine green after peripheral intravenous injection is a simple, effective, and safe method to locate the nodule. This offers surgeons the ease of localization through direct indocyanine green fluorescence imaging, and it can be used as an effective alternative to other placement methods of locating pulmonary nodules.

## Introduction

With the popularization of chest computed tomography (CT), more lung cancer patients are detected at an early stage. At present, thoracoscopic cuneiform resection and segmental resection are the most commonly used surgical methods for early lung cancer (1). Minimally invasive surgery, including video-assisted thoracoscopic surgery (VATS), has been considered ideal for the removal of small nodules because it has the lowest postoperative morbidity and mortality, is less painful, and offers a better quality of life (2, 3). Owing to the visual and tactile limitations of thoracoscopic surgery, intraoperative identification of pulmonary nodules, especially solid or deep nodules, is challenging when there is no change in visceral pleura (4–6). In order to accurately remove pulmonary nodules and reduce lung loss, thoracic surgeons have adopted various positioning methods in clinical practice to overcome the limitations of thoracoscopic surgery (7).

Inadequate nodule location may lead to prolonged surgery for nodular search or lead to conversion to unplanned thoracotomy or extended excision (5, 6, 8).With the current shift to minimally invasive surgery, the location of intraoperative nodules has become a key challenge. Preoperative localization techniques have been introduced as a way to improve the success rate of VATS and prevent unnecessary thoracotomy (5, 6).

At present, the most widely used methods for locating pulmonary nodules in clinical practice are placement, including hookwire, microcoil, trailing positioning hooks, and liquid materials (indocyanine green (9), etc.), all of which have a high success rate and certain complications. Complications may lead to patient discomfort; metal tag dislocation, pneumothorax, and hemoptysis may occur; and placement positioning also has the disadvantage of not delaying surgery. Other localization methods include broncho-assisted localization technology and intraoperative anatomical localization, which have certain advantages and disadvantages.

Based on previous studies on the localization of indocyanine green and the pharmacotoxicology of indocyanine green, we tried to develop a new localization method. The objective of this study was to determine the feasibility and safety of ICG fluorescence localization of peripheral blood pathways and near-infrared (NIR) fluorescence thoracoscopic resection of pulmonary nodules.

## Materials and methods

### Study plan and patient groups

This clinical trial used ICG peripheral intravenous puncture to cause small bleeding of lung tissue for local staining purposes. Thoracoscopic surgery was performed using near-infrared (NIR) fluorescent thoracoscopy [PC9000, Stryker (Beijing) Medical Device Co., Ltd., USA]. The study was approved by the Ethics Committee of Shenyang Chest Hospital, and all patients gave informed consent.

The inclusion criteria for our study were undiagnosed patients with non-solid pulmonary nodules no larger than 2 cm. According to C/T, it can be divided into pure ground glass nodules and partial solid nodules [(<0% C/T ratio ≤50%) and (<50% C/T ratio <100%)]. C/T is defined as the ratio of the maximum diameter of solid component of pulmonary nodules to the maximum diameter of the ground glass component. Sublobectomy was performed after chest CT 3D reconstruction and preliminary positioning. Patients with suspected preoperative lymph node metastasis underwent preoperative endobronchial ultrasound transbronchial aspiration to confirm that the mediastinal lymph nodes were clear of disease (10). Our exclusion criteria were (1) sublobectomy is not suitable after imaging evaluation, and (2) patients with small pulmonary nodules who have developed lymph node or distant metastasis.

### Materials

The ICG (H20055881, Dandong Medical Innovation Pharmaceutical Co., Ltd.) is a water-soluble anionic, amphiphilic NIR fluorophore with an excitation wavelength of 790 nm, an emission wavelength of 830 nm, and a molecular weight of 774.96 kDa. Allergy to this preparation is rare; pregnant women and patients with a history of iodine allergy should be noted.

A dose of 10 mL of ICG at a concentration of 0.3 mg/kg was injected into the body through a peripheral vein. After a single successful puncture was confirmed by CT, the puncture needle was pulled out. The dose and concentration of ICG were determined based on previous studies such as the appearance of indocyanine green in the intersegmental plane of segmental lungectomy to exhibit higher fluorescence intensity. ICG fluorescence in lung tissue was captured intraoperatively by NIR thoracoscopy. The fluorescent thoracoscope is a rigid thoracoscope produced by Stryker Company, USA. The optical system has a 30° view angle, transmitting both visible light and 808 nm ± 5nm NIR laser.

### Procedure

**Figure 1:**
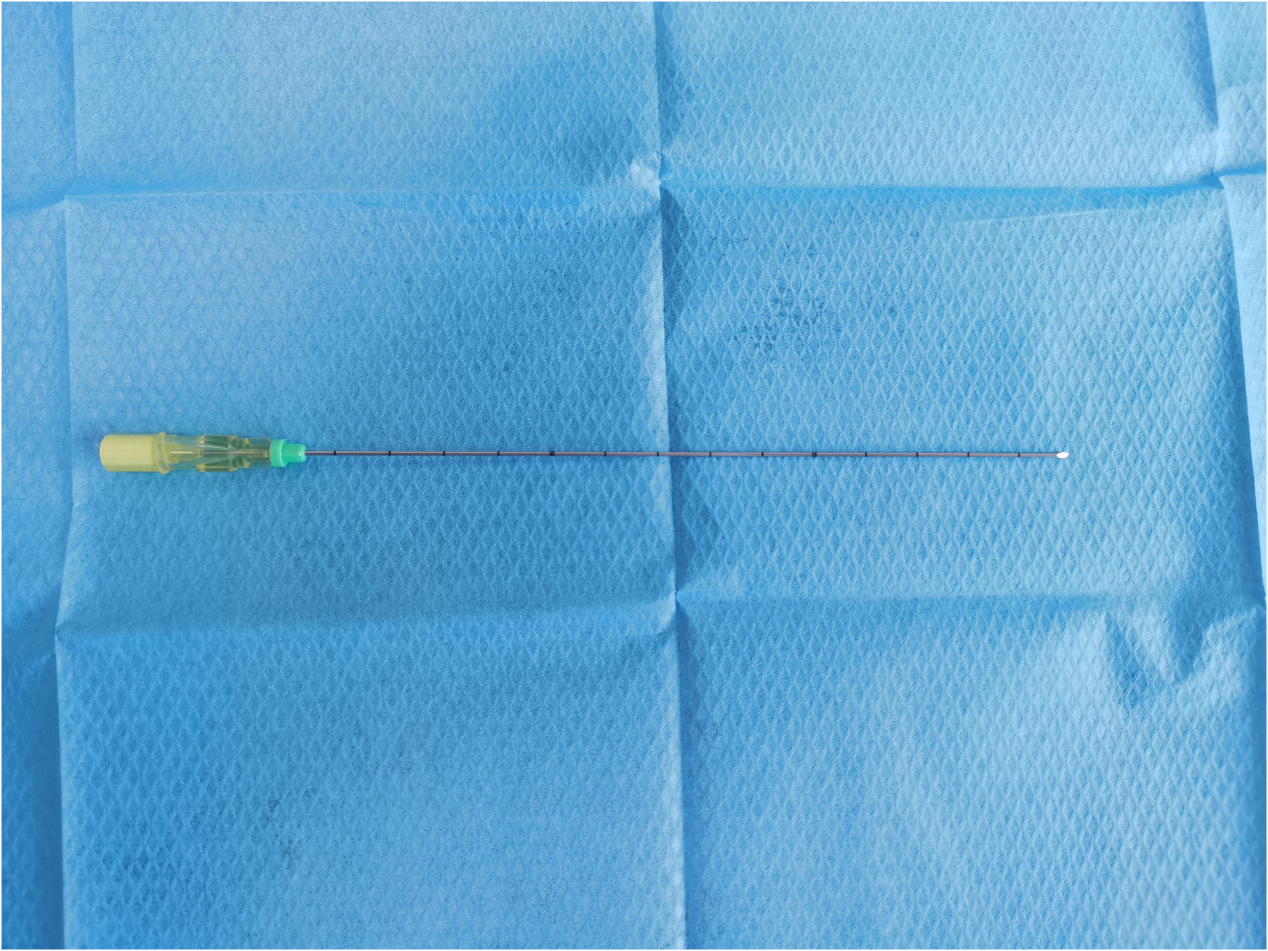
Puncture needle.

The objective of this study was to determine the feasibility of using peripheral intravenous ICG fluorescence to locate nodules. All surgical procedures were performed in the catheterization lab and multifunctional operating room of Shenyang Chest Hospital with equipment including a CT scanner (GE Optima ct680, USA) and a 20-gauge needle (20G*150 mm, Hakko Co., Ltd., Chikuma-Shi Nagano, Japan) (**Figure 1**).

**Figure 2:**
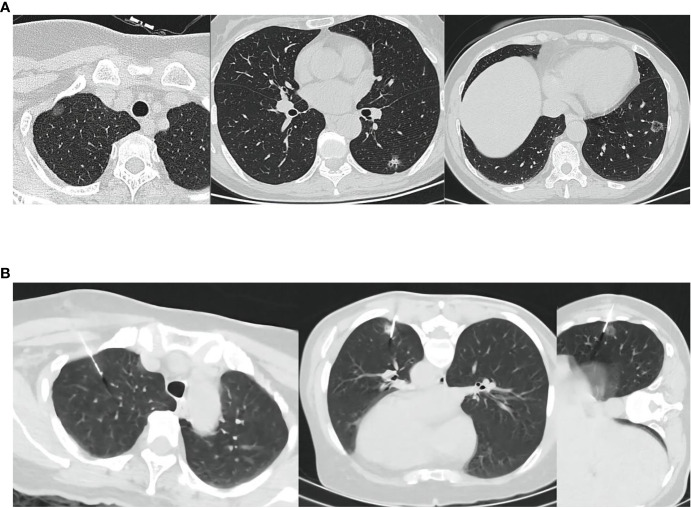
**(A)** The location of the lesion shown on CT. **(B)** Indocyanine green was injected intravenously and punctured under CT. CT, Computed tomography; ICG, indocyanine green.

First, after the location of pulmonary nodules was confirmed under CT, routine disinfection was performed, sterile cloth was applied, and, after local infiltration and anesthesia, the puncture needle was inserted into the chest wall near the pulmonary nodules (without penetrating the parietal pleura). After the angle and depth were adjusted, 0.3 mg/kg indocyanine green (diluted to 0.25 mg/mL) was injected intravenously into the peripheral area. We chose to administer indocyanine green intravenously 10–15 s later for lung puncture. The puncture needle is inserted into the lung approximately 1 cm deep near the small lung nodule that needs to be treated. According to the results of the study on the pulmonary intersegment plane displayed after intravenous injection of indocyanine green, median times from the intravenous injection of ICG to intersegmental plane detection, maximum visualization, and disappearance were 15 s (range, 3–60 s), 40 s (range, 10–155 s), and 90 s (range, 30–420 s), respectively (11, 12).Therefore, we chose to perform puncture 10–15 s after indocyanine green injection. After ICG was intravenously injected into the body, indocyanine green immediately bound to plasma protein. After puncture, small blood vessels in pleura and lung burst, causing blood to be exposed and coagulated. Indocyanine green was coagulated in plasma protein around the puncture, thus enabling fluorescence thoracoscopy to detect the location of a small nodule. In other words, the CT scan confirms the position of the puncture needle in the lung (near the nodule to be located) and the post-insertion depth (**Figure 2**), a single puncture is done within 15 to 40 s of the ICG injection, and the puncture needle could be pulled out and bandaged, followed by transfer to the operating room.

**Figure 3:**
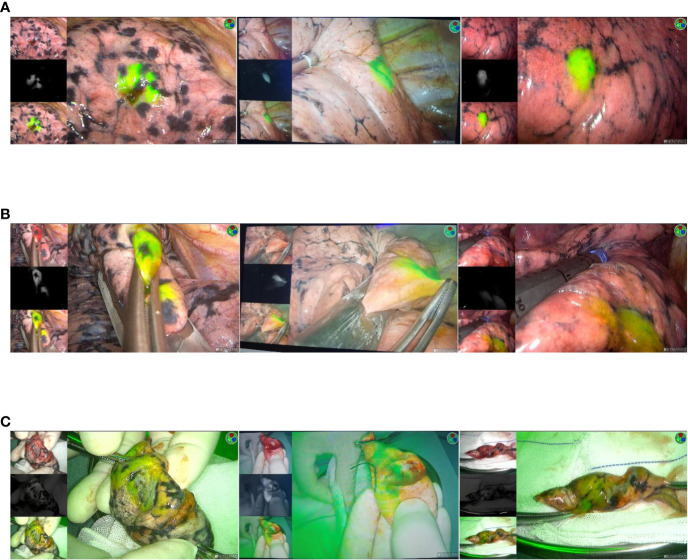
**(A)** Intraoperative imaging using a NIR thoracoscope. The location of the lesion is dsiplayed. **(B)** Thoracoscopic instrument was used to cut the lesion. **(C)** Resected specimen.

After initiation of VATS, the nodule was located by ICG fluorescence using NIR thoracoscopy (**Figure 3A**). If a wedge resection is to be performed, the lung is clamped using a thoracoscopic instrument to set up a sufficient margin for the anticipated excision (**Figure 3B**). If a pulmonary segment resection is to be performed, the relevant anatomy will be performed. Finally, after the lesion was removed, the specimen was cut along the maximum diameter of the lung tumor for thoracoscopic observation with NIR (**Figure 3C**), and the surgical margin was visually observed to ensure that the incision margin was larger than 2 cm or larger than the tumor diameter. Frozen section pathology was used to confirm that the nodules were removed and all surgical margins were negative. If the surgical margin is macroscopically close to the tumor, excision is performed and pathological evaluation is performed. All tumors were reviewed by a specialized pulmonologist.

### Outcome measures

The primary objective of this study was to demonstrate the feasibility and safety of our peri-intravenous ICG instant pulmonary puncture and NIR video-assisted fluorescence localization system. Through this study, we hope to demonstrate that this technique is superior to the traditional CT-guided placement localization method.

## Result

From August 2021 to April 2022, 18 patients with non-solid small nodules around the lung and suspected malignant tumors were treated with CT-guided ICG lung puncture via the peripheral vein and NIR image-guided VATS resection. All procedures described in this trial were sublobectomies. **Table 1** summarizes the characteristics of the patients. Demographic information is shown in **Table 2**.

**Table 1:** Patient characteristics and tumor data.

Patient characteristics and tumor variables	Value (*n* = 18)
Median age (range), years	66.5 (52–75)
Gender	
Male	10
Female	8
Median CT nodule size (range), cm	1.1 (0.5–2.0)
Median CT nodule depth from surface (range), cm	1.6 (0.1–4.6)
Nodule location	
Right upper lobe	6
Right middle lobe	1
Right lower lobe	4
Left upper lobe	3
Left lower lobe	4
CT findings	
Pure GGN	5
Part solid GGN (<0% C/T ratio ≤ 50%)	5
Part solid GGN (<50%C/T ratio < 100%)	4
Type of surgery	
Segmentectomy	6
Wedge resection	12
Pathologic diagnosis	
Adenocarcinoma	17
Adenocarcinoma *in situ*	6
Minimally invasive adenocarcinoma	8
Invasive adenocarcinoma	1
Adenocarcinoma with adherent growth pattern	2
Benign lesion	1

CT, computed tomography; GGN, ground glass nodule.

**Table 2:** Study outcome.

Case no.	ICG Fluorescence	Gender	Age (years)	Tumor location	Image finding	Tumor size (cm)	C/T ratio (%)	Tumor depth (cm)	Pathologic diagnosis
1	Clear	F	60	RUL	Part solid GGN (<50% C/T ratio <100%)	0.8	60%	1.6	Adenocarcinoma with adherent growth pattern
2	Clear	F	65	RLL	Pure GGN	0.5	0	1.2	AIS
3	Clear	M	67	LUL	Part solid GGN (<0% C/T ratio ≤50%)	1.3	21%	2.1	MIA
4	Clear	M	71	LUL	Part solid GGN (<0% C/T ratio ≤50%)	1.6	16%	4.6	AIS
5	Clear	M	75	RUL	Part solid GGN (<0% C/T ratio ≤50%)	1.1	42%	1.7	AIS
6	Clear	F	62	RLL	Pure GGN	0.6	0	0.1	MIA
7	Clear	F	58	LLL	Part solid GGN (<50% C/T ratio <100%)	1.9	73%	0.9	MIA
8	Clear	F	66	RML	Part solid GGN (<0% C/T ratio ≤50%)	0.5	15%	1.4	MIA
9	Clear	M	60	RUL	Pure GGN	1.3	0	1.1	AIS
10	Clear	M	73	LLL	Part solid GGN (<50% C/T ratio <100%)	1.0	77%	0.3	IA
11	Clear	M	59	LLL	Part solid GGN (<0% C/T ratio ≤50%)	1.2	15%	0.4	MIA
12	Clear	F	66	RUL	Part solid GGN (<0% C/T ratio ≤50%)	2.0	10%	3.7	AIS
13	Clear	M	52	LUL	Part solid GGN (<0% C/T ratio ≤50%)	1.7	9%	2.0	MIA
14	Clear	F	58	RLL	Part solid GGN (<0% C/T ratio ≤50%)	0.9	20%	2.2	MIA
15	Clear	M	64	RUL	Part solid GGN (<0% C/T ratio ≤50%)	1.3	22%	1.7	AIS
16	Clear	M	69	RUL	Pure GGN	0.8	0	1.9	Benign (AAH)
17	Too large	F	58	RLL	Pure GGN	2	0	1.0	MIA
18	Unclear	M	71	LLL	Part solid GGN (<50% C/T ratio <100%)	1.8	87%	0.9	Adenocarcinoma with adherent growth pattern

ICG, indocyanine green; C/T, consolidation/tumor; F, female; M, male; LUL, left upper lobe; RML, right middle lobe; LLL, left lower lobe; RLL, right lower lobe; MIA, minimally invasive adenocarcinoma; RUL, right upper lobe; GGN, ground glass nodule; AIS, adenocarcinoma in situ; IA, invasive adenocarcinoma; MIA, minimally invasive adenocarcinoma; AAH, atypical adenomatous hyperplasia.

There were 8 female patients and 10 male patients, with a mean age of 64.5 years (range, 52–75 years). There were four mainly solid partially solid ground glass nodules on CT (<50% C/T ratio <100%), nine partially solid ground glass nodules (<0% C/T ratio ≤50%), and five pure ground glass nodules (C/T ratio = 0%).The median CT tumor size was 1.2 cm (range, 0.5–2 cm). The median distance from the pleural surface to the nodal surface was 1.6 cm (range, 0.1–4.6 cm). The mean CT-guided intervention time was 12 min (range, 7–24 min), and VATS wedge resection and segmentectomy procedural time was 67 min (range, 26–147 min).

All patients were successfully injected with ICG via the peripheral vein. During the operation, ICG fluorescence was clearly recognized in all patients except one patient (17/18:94.4%). Nodules were successfully located and resected by thoracoscopic NIR in 17 patients. One patient had a 1.8-cm partially solid nodule dominated by ground glass located 1 cm from the pleura. ICG fluorescence was not detected; thus, preoperative CT three-dimensional reconstruction combined with anatomical localization was used for surgery (Case 18). Among the 17 cases, 1 showed a wide range of fluorescence under a microscope, but did not affect the operation. Of the 18 cases, 12 cases were wedge-shaped resection and 6 cases were pulmonary segment resection. After surgical excision, we confirmed the lesion margin and NIR fluorescence in excised specimens. In all cases, the target nodules were successfully excised and surgical margins were negative. There were no adverse reactions associated with ICG injection. No perioperative or postoperative complications occurred. All patients were discharged from the hospital without any surgical complications. The final diagnosis of 20 patients included 17 adenocarcinomas (94.4%) and 1 benign lesion (5.6%). Intraoperative lymph node sampling was performed in five patients to confirm negative lymph node metastasis. Among the 17 primary lung adenocarcinomas, 6 were *in situ*, 8 were microinvasive, 1 was adenocarcinoma, and 2 were adherent growth adenocarcinoma (**Table 1**). Surgical margins of all excised specimens were microscopically negative.

## Discussion

ICG is approved as an intravenous drug for indications and uses such as determination of cardiac output, liver function, and liver blood flow, and for ophthalmology and cardiovascular angiography. ICG is also used to detect sentinel lymph nodes in skin melanoma (13–15), breast cancer (16–18), gynecological cancer (19), head and neck cancer (20), and lung cancer (21).

After intravenous injection, ICG immediately binds to plasma proteins and rapidly distributes in blood vessels throughout the body along with blood circulation. At this time, if there is bleeding, ICG will be fixed to the bleeding site by the protein in the coagulated blood, and ICG fluorescence will be identified under thoracoscopy. Based on this principle, we started the study. In this study, after the location of the nodule was confirmed by CT, ICG was injected intravenously and the puncture needle was punctured near the nodule in the lung. We knew that puncture would cause a small amount of bleeding at the puncture site near the nodule, and the small amount of bleeding would solidify the plasma protein containing ICG near the nodule. The plasma protein containing ICG could be observed immobilized by fluorescence thoracoscopy, so as to achieve the purpose of positioning. NIR thoracoscopic technology was used to display and overlay both fluorescent and white light images during VATS [PC9000, Stryker (Beijing) Medical Device Co., Ltd., USA]. The use of ICG fluorescence for NIR imaging has advantages over other techniques. It uses three types of laser and deep tissue excitation of ICG, and the penetrating properties of the light emitted by NIR facilitate the visualization of small amounts of ICG in the lung parenchyma. It is safe for humans.

In our trial, 18 patients with isolated pulmonary nodules underwent VATS resection after peripheral intravenous ICG. NIR signals detected 94.4% of tumors without complications.

In 17 of the 18 patients, ICG fluorescence penetrating lung tissue was successfully captured by a NIR thoracoscope and changes in lung surface color were observed. Unlike white light endoscopy, which detects color dyes, the specific wavelength of ICG fluorescence is always detectable regardless of any change in the color or texture of the visceral pleura. ICG fluorescence imaging is easy to observe, real-time, and intuitive to the observer. This feature gives ICG an advantage over other implantable markers that must be removed with pulmonary nodules during surgery. Studies such as Hideki Ujiie et al. have shown excellent results for pulmonary surface injection of ICG localization (9), as well as other liquid markers such as radioactive tracers, iodine, and methylamine (22).

As for the indication of pulmonary nodule localization, we believe that our study is more extensive than other indications of implantable localization, and all pulmonary nodule localization and even smaller nodule are suitable, due to the simplicity of this technique, because a single puncture is more convenient, the operation time is shorter, and there are no more complications compared to other methods.

Severe air embolism in percutaneous CT-guided lung biopsies should be considered as a potential complication of percutaneous marker insertion (23). Dislocation of wires and diffusion of dyes and contrast agents are some of the technical challenges that have been reported (6). With the advent of new technologies such as navigational bronchoscopy, localization may seem less invasive, but this approach still requires full-dose thin-slice CT scans to construct navigational as well as fluoroscopic images during bronchoscopy. Therefore, the cost cannot be ignored, and not all intrapulmonary lesions are available. This localization method does not add more cost, but requires surgery under a fluorescence thoracoscope, which is even more economical than other implantable locating methods.

In contrast to the inadvisability of the implant in insertional positioning, intravenous ICG can allow surgical delay or cancel due to specific conditions. Because ICG binds to plasma proteins, the ICG display will disappear after the absorption of the possibly coagulated plasma proteins. If the operation is cancelled, this positioning technique will not cause subsequent complications.

In addition, the insertion or injection of liquid materials requires a thicker puncture needle, usually 18G or 19G, while our puncture needle is 20G, which is less likely to cause bleeding or pneumothorax and only requires a small amount of bleeding on the lung surface after puncture. However, the simple operation of a single puncture does not require placement of positioning markers, which reduces the probability of air embolization and greatly shortens the positioning operation time. No obvious and clear fluorescence was observed in one patient under fluorescence, which we considered might be related to emphysema of the patient, which resulted in reduced blood vessels in local pulmonary parenchyma, resulting in no bleeding or an extremely small amount of blood, which failed to develop. The fluorescence imaging of another patient was larger than expected, which must be caused by excessive bleeding. This suggested that we should avoid vessels visible by CT as much as possible for puncture, because it might lead to much more blood loss than we needed, resulting in excessive fluorescence imaging.

Based on our study, guided pulmonary puncture followed by peripheral venous ICG injection and intraoperative NIR localization of small nodules is a safe, simple, and feasible method in minimally invasive surgery. It provides the thoracic surgeon with the convenience of positioning by direct ICG fluorescence imaging with localization accuracy, safety, and ease of operation comparable to any other placement localization method.

We acknowledge that the current approach has limitations and therefore there is room for technical improvement. In this study, two cases did not achieve the desired positioning effect. Emphysema lesions may affect the accuracy of this localization method, and ICG fluorescence display has been shown to be limited in lungs with air volume retention (24). For small nodules located deep in the lung parenchyma, this localization technique can only be located on the lung surface. We believe that this positioning can confirm a plane that guides the incisal margin of sublobectomy to ensure adequate incisal margin.

Vessels visible on subpleural CT images should be avoided. Studies have shown that tissue penetration of NIR fluorescence images is limited at depths of more than 24 mm (24). Therefore, lesions deeper from the pleura can only be located on the lung surface, and these factors should be taken into account when selecting ICG location for peripheral intravenous injection. In the present study, we used a CT scanner for localization in the cath lab. After the localization, patients needed to be transported to the operating room, which increased the risk of transportation. If possible, it is more reasonable to locate and operate in the digital integrated operating room of the future.

## Conclusions

We describe a new, more maneuverable localization method using NIR thoracoscopy and ICG fluorescence for image-guided localization and minimally invasive resection of pulmonary nodules. This is a safe and simple minimally invasive method for locating and removing pulmonary nodules. It is an effective alternative to other implantable positioning methods.

## Data availability statement

The original contributions presented in the study are included in the article/supplementary material. Further inquiries can be directed to the corresponding author.

## Ethics statement

Written informed consent was obtained from the individuals for the publication of any potentially identifiable images or data included in this article.

## Author contributions

ZL: Conceptualization, Formal Analysis, Writing – original draft. MX: Data curation, Formal Analysis, Writing – review & editing. CL: Data curation, Investigation, Software, Writing – review & editing. LX: Formal Analysis, Methodology, Software, Writing – review & editing. TW: Formal Analysis, Investigation, Writing – review & editing. YR: Conceptualization, Project administration, Supervision, Writing – review & editing.
